# Excessive folate synthesis limits lifespan in the *C. elegans*: *E. coli *aging model

**DOI:** 10.1186/1741-7007-10-67

**Published:** 2012-07-31

**Authors:** Bhupinder Virk, Gonçalo Correia, David P Dixon, Inna Feyst, Jie Jia, Nikolin Oberleitner, Zoe Briggs, Emily Hodge, Robert Edwards, John Ward, David Gems, David Weinkove

**Affiliations:** 1 School of Biological and Biomedical Sciences, Durham University, South Road, Durham, DH1 3LE, UK; 2 Biophysical Sciences Institute, Durham University, South Road, Durham, DH1 3LE, UK; 3 Faculty of Science, University of Lisbon, Campo Grande, 1749-016, Lisbon, Portugal; 4 Department of Structural and Molecular Biology, ISMB, University College London, London WC1E 6BT, UK; 5 Institute of Healthy Ageing and Department of Genetics, Evolution and Environment, University College London, London WC1E 6BT, UK

**Keywords:** aging, microbes, folate, *C. elegans*, *E. coli*

## Abstract

**Background:**

Gut microbes influence animal health and thus, are potential targets for interventions that slow aging. Live *E. coli *provides the nematode worm *Caenorhabditis elegans *with vital micronutrients, such as folates that cannot be synthesized by animals. However, the microbe also limits *C. elegans *lifespan. Understanding these interactions may shed light on how intestinal microbes influence mammalian aging.

**Results:**

Serendipitously, we isolated an *E. coli *mutant that slows *C. elegans *aging. We identified the disrupted gene to be *aroD*, which is required to synthesize aromatic compounds in the microbe. Adding back aromatic compounds to the media revealed that the increased *C. elegans *lifespan was caused by decreased availability of para-aminobenzoic acid, a precursor to folate. Consistent with this result, inhibition of folate synthesis by sulfamethoxazole, a sulfonamide, led to a dose-dependent increase in *C. elegans *lifespan. As expected, these treatments caused a decrease in bacterial and worm folate levels, as measured by mass spectrometry of intact folates. The folate cycle is essential for cellular biosynthesis. However, bacterial proliferation and *C. elegans *growth and reproduction were unaffected under the conditions that increased lifespan.

**Conclusions:**

In this animal:microbe system, folates are in excess of that required for biosynthesis. This study suggests that microbial folate synthesis is a pharmacologically accessible target to slow animal aging without detrimental effects.

## Background

The microbial flora found in the gastrointestinal tract influences human metabolism and physiology and is thus likely to impact aging [1,2]. Changes in the microbial flora are associated with obesity [3,4] and microbial metabolism may influence cardiovascular disease [5]. In the *C. elegans *model, the *E. coli *foodstuff must be alive, but not necessarily able to divide, for the worm to achieve maximal growth and reproduction [6-8], suggesting that microbial metabolic activity is required for optimal *C. elegans *nutrition. Dietary restriction of *C. elegans *by limiting *E. coli *availability extends lifespan but the mechanisms involved remain unclear [9,10]. Treating *E. coli *with antibiotics that either stop proliferation or kill the bacteria increases *C. elegans *lifespan [11,12] and it has been previously suggested the mutants in *E. coli *genes that disrupt ubiquinone synthesis increase *C. elegans *lifespan by blocking bacterial respiration [13]. However, slowing or stopping bacterial growth is not a viable starting point to treat healthy microbiota. Lipopolysaccharide structures on the *E. coli *cell surface explain bacterial strain-specific effects on *C. elegans *lifespan and interactions with the *C. elegans *sensory system but do not provide a clear route to slow aging [14]. Here we explore the *C. elegans/E. coli *interaction further and identify microbial folate synthesis as a specific target to slow animal aging pharmacologically without cost to the microbe or animal.

## Results and Discussion

### A spontaneous mutation in the *E. coli *gene *aroD *increases *C. elegans *lifespan

**Figure 1:**
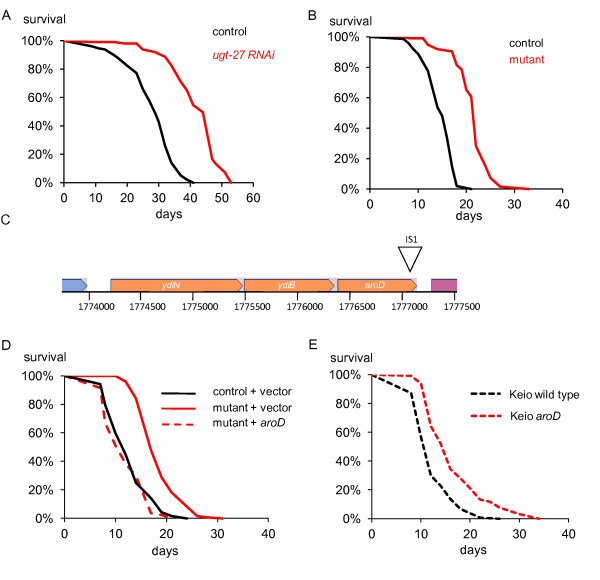
**Identification of a spontaneous *E. coli *mutant that extends lifespan**. **A**) Survival curves of *rrf-3(pk1426); daf-2(m577) *animals at 25°C on the control strain (n = 201): HT115(DE3) containing the empty vector L4440 and a strain, containing the plasmid for the *ugt-27 *gene, that caused a 50% increase in lifespan (n = 92, *P *= < 0.0001). **B**) Survival of *rrf-3 *(25°C) on the mutant strain from which the *ugt-27 *has been lost (n = 68) compared with the control strain in which the L4440 empty vector has been lost (n = 62). Increase in lifespan = 49.6% (*P = *< 0.0001). **C**) Position of the IS1 transposon insertion at position 1777116 on the *E. coli *K12 W3110 chromosome [44] (diagram based on EcoCyc.org [45]). As the insertion is at nucleotide 717 of the *aroD *open reading frame, the allele is designated *aroD717::IS*1. **D**) The lifespan effect of the mutant bacteria is rescued by the plasmid containing *aroD. glp-4(bn2) *animals were raised on the *aroD *mutant until L4 (15°C) and then transferred to the *aroD *mutant + pMMB67EH vector (n = 126), *aroD *mutant + pMMB67EH plasmid containing *aroD *region (n = 85), wild type bacteria + vector (n = 126) (25°C). **E**) Worms maintained on the *aroD *deletion mutant from the Keio collection (n = 128) show an extended lifespan compared to wild type (n = 131). *P *= < 0.001.

While performing lifespan experiments using RNA interference (RNAi) by feeding, we discovered an *E. coli *HT115(DE3) RNAi strain, for the *C. elegans ugt-27 *gene, that causes a substantial (30 to 50%) increase in lifespan of the long-lived *daf-2 *mutant of *C. elegans *compared to animals maintained on the HT115(DE3) control (Figure 1A). The strain also extends the lifespan of wild type *C. elegans*, and a *daf-16 *mutant lacking the FOXO transcription factor required for *daf-2 *mutant longevity (Additional file 1). Surprisingly, the lifespan increase persisted once the RNAi plasmid was lost, implicating a spontaneous mutation of the *E. coli *strain as the causative factor (Figure 1B). Consistent with this conclusion, fresh HT115(DE3) bacteria transformed with the *ugt-27 *RNAi plasmid had no effect on lifespan (Additional file 2). Unlike previously identified *E. coli *mutants that result in extended *C. elegans *lifespan [13,15], this mutant *E. coli *strain was able to respire but unable to grow on minimal media. This auxotrophy allowed us to perform a plasmid complementation screen leading to the identification of an IS1 transposon insertion in the gene *aroD *(Figure 1C). Plasmid rescue with *aroD *confirmed that mutation of this gene increased *C. elegans *lifespan (Figure 1D). A deletion mutation of *aroD *from the Keio strain collection also showed an increased lifespan compared to animals fed the control strain [16]. (Additional file 1, Figure 1E), demonstrating that the effect was not allele- or strain-specific.

### Folate synthesis is the limiting factor that causes the *aroD *mutant to increase *C. elegans *lifespan

**Figure 2:**
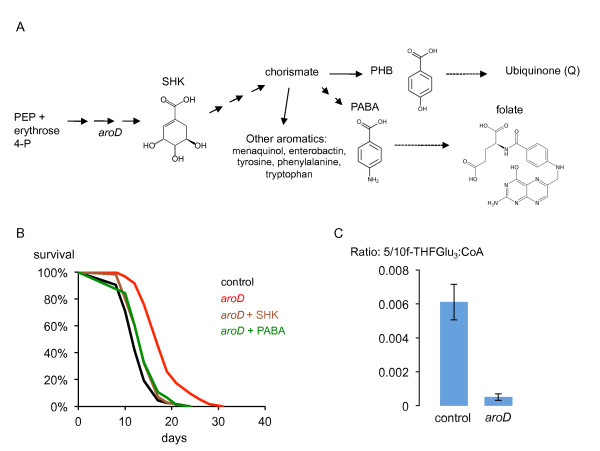
**Decreased folate synthesis explained the lifespan increase caused by the *aroD *mutant**. **A**) Schematic of the shikimic acid and folate synthesis pathways. Solid arrows represent single enzymatic steps. Dashed arrows represent multiple steps. PEP, phosphoenolpyruvate; SHK, shikimic acid; PABA, para-aminobenzoic acid. **B**) Shikimic acid and PABA reverse the lifespan increase caused by the *aroD *mutation. *glp-4(bn2) *animals were raised on the *aroD *mutant until L4 (15°C) and then transferred to the *aroD *mutant (n = 116), wild type control (n = 116), *aroD *+ SHK (n = 86), *aroD *+ PABA (n = 76), (25°C). All supplements at 40 μg/ml. *aroD *+ PABA vs *aroD*, *P *= < 0.0001; *aroD *+ SHK vs *aroD*, *P *= < 0.0001. **C**) Levels of formylTHFGlu_3 _as detected by LC-MS are decreased in the *aroD *mutant compared to the wild type HT115(DE3). Data from two biological replicates.

The *aroD *gene encodes the enzyme 3-dehydroquinate dehydratase, a core component of the shikimic acid pathway that produces chorismate, a precursor to all aromatic compounds in the bacterial cell (Figure 2A) [17,18]. Consistent with the involvement of this pathway, supplementation of the media with shikimic acid causes the lifespan of *C. elegans *on *aroD *mutant bacteria to revert to normal (Figure 2B). The *aroD *mutant can grow on the peptone-based media used in *C. elegans *studies, so the media must be able to provide either all the essential aromatic compounds needed for growth or the relevant precursors. To test whether the lifespan effect was caused by one of these compounds being present in limiting amounts, we added back compounds known to support growth of *aro *mutants: the aromatic amino acids, the folate precursor para-aminobenzoic acid (PABA) and the ubiquinone precursor para-hydroxybenzoic acid (PHB) [17]. Of these, only PABA reversed the lifespan increase completely, suggesting that a decrease in bacterial folate synthesis in the *aroD *mutant is the major cause of the increased *C. elegans *lifespan (Figures 2B; Additional file 3, Figure A; Additional file 3, Figure C; Additional file 1). PABA supplementation had no effect on *C. elegans *maintained on the control HT115(DE3) strain or on the extended lifespan of worms on the Q-deficient *ubiG *mutant bacteria ruling out a toxic effect of PABA (Additional file 3, Figure B). An alternate pathway for ubiquinone synthesis using PABA instead of PHB has been shown in *Saccharomyces cerevisiae *[19,20] but as PHB has no effect on lifespan (Additional file 3, Figure C) we think it unlikely that ubiquinone synthesis is the limiting factor in the *aroD *mutant. Folates are needed in all cells for biosynthesis. Generation of purines, pyrimidines, certain amino acids and methyl donors depends on cycling between the various folate species: dihydrofolate (DHF), tetrahydrofolate (THF), 10-formyl THF, 5,10-methenyl THF, 5,10-methylene THF and 5-methyl THF [21]. In addition, over 100 possible folate species result from further derivatization with up to 8 glutamate residues. To understand the effect of the *aroD *mutation on bacterial folates, we used liquid chromatography coupled to mass spectrometry (LC-MS) to detect individual folate species [21,22]. The most abundant folate species detected in *E. coli *grown as lawns on NGM agar corresponded to formyl THFGlu_3_. We found that the *aroD *mutation in HT115(DE3) caused a large decrease in the detectable levels of this folate and other detectable folate species (Figure 2C, Additional file 4), confirming the effect of the mutation on bacterial folates. Folic acid cannot be taken up directly by *E. coli*. However, adding back folic acid to the media resulted in a partial suppression of the increase in lifespan, probably because folic acid led to restoration of folate synthesis in the *aroD *mutant bacteria (Additional file 5). It has been shown that *E. coli *can use a breakdown product of folic acid to make PABA [23].

### Pharmacological inhibition of *E. coli *folate synthesis increases *C. elegans *lifespan

**Figure 3:**
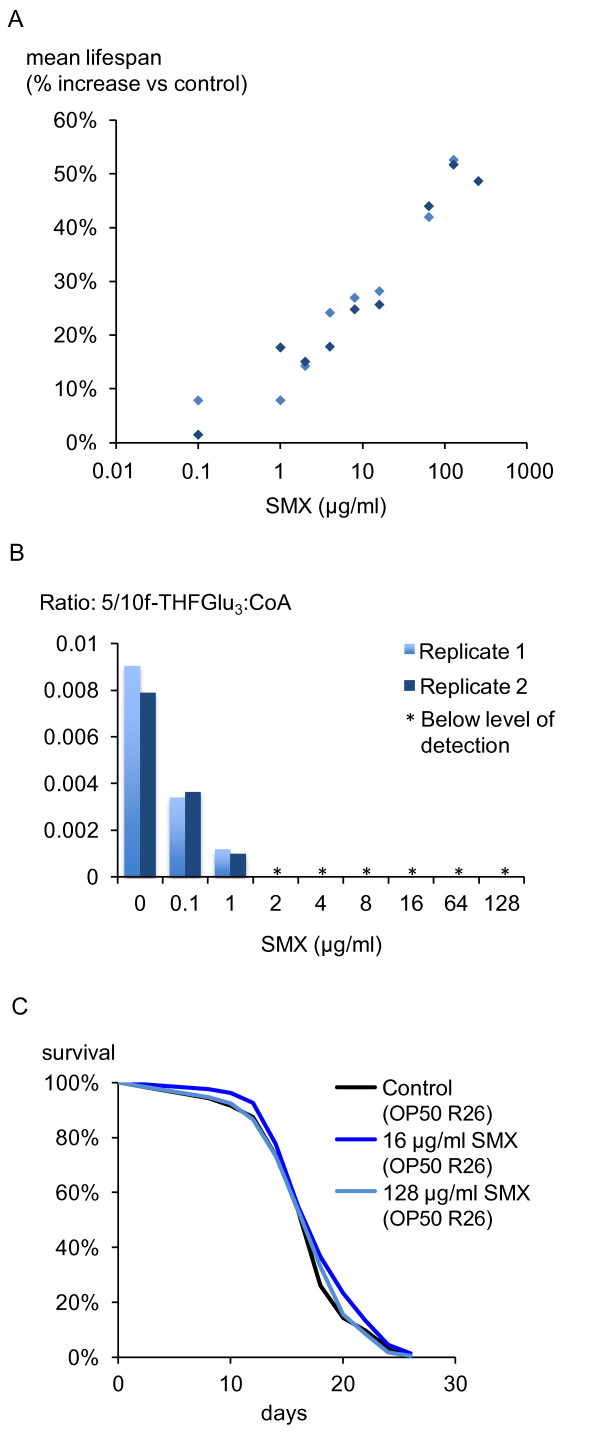
**Inhibition of bacterial folate synthesis causes an increase in *C. elegans *lifespan**. **A**) Treatment of OP50 with various concentrations of SMX increases mean *C. elegans *lifespan by the indicated percentage. Control (n = 102, 191), 0.1 μg/ml SMX (n = 139, 221), 1 μg/ml (n = 154, 229), 2 μg/ml (n = 152, 210), 4 μg/ml (n = 161, 226), 8 μg/ml (n = 146, 224), 16 μg/ml (n = 176, 226), 64 μg/ml (n = 235, 238), 128 μg/ml (n = 229, 230). 256 μg/ml (n = 253). **B**) SMX treatment decreases levels of formylTHFGlu_3 _in *E. coli *OP50 until it becomes undetectable at 2 μg/ml. * = below the level of detection. Two biological replicates are shown. **C**) The lifespan increase induced by 16 and 128 μg/ml SMX is eliminated when worms are maintained on OP50 containing the R26 plasmid that confers sulfonamide resistance. Control (n = 224), 16 μg/ml SMX (n = 218), 128 μg/ml SMX (n = 215).

To test further the impact of microbial folate synthesis on *C. elegans *aging, we employed sulfamethoxazole (SMX), a sulfonamide drug that blocks folate synthesis by competing with PABA for the enzyme dihydropteroate synthase [24]. Addition of SMX to the media caused a dose-dependent increase in the lifespan of worms maintained on *E. coli *OP50, the strain used in most *C. elegans *studies (Figure 3A), with 2 μg/ml being the minimal dose that gave a reproducible and statistically significant effect. With increasing drug concentration, the relationship between mean lifespan and log[SMX] is approximately linear until 128 μg/ml, the concentration of SMX that consistently produced the highest increase in mean lifespan (See Additional file 1 for the full data set). This linear relationship suggests a dose response that is pharmacologically amenable. Addition of PABA reverses the increase in lifespan, consistent with folate synthesis being the relevant target of SMX (Additional file 6). To assess the impact of SMX on bacterial folates, we measured formyl THFGlu_3_. Starting at a concentration of 0.1 μg/ml, SMX reduced the levels of formyl THFGlu_3 _in OP50 such that at a dose of 2 μg/ml SMX it was effectively below the level of detection (Figure 3B; Additional file 4, Figure B). To determine whether SMX increased lifespan through any direct effects on the worm and/or any non-specific targets in *E. coli*, we performed lifespan experiments with a sulfonamide-resistant strain of OP50, which contains the multiresistance plasmid R26 encoding a drug-insensitive allele of dihydropteroate synthase [25]. Under these conditions, SMX had no effect on *C. elegans *lifespan (Figure 3C), demonstrating that SMX acts through inhibition of bacterial folate synthesis to increase *C. elegans *lifespan.

### SMX has no effect on bacterial growth or viability

**Figure 4:**
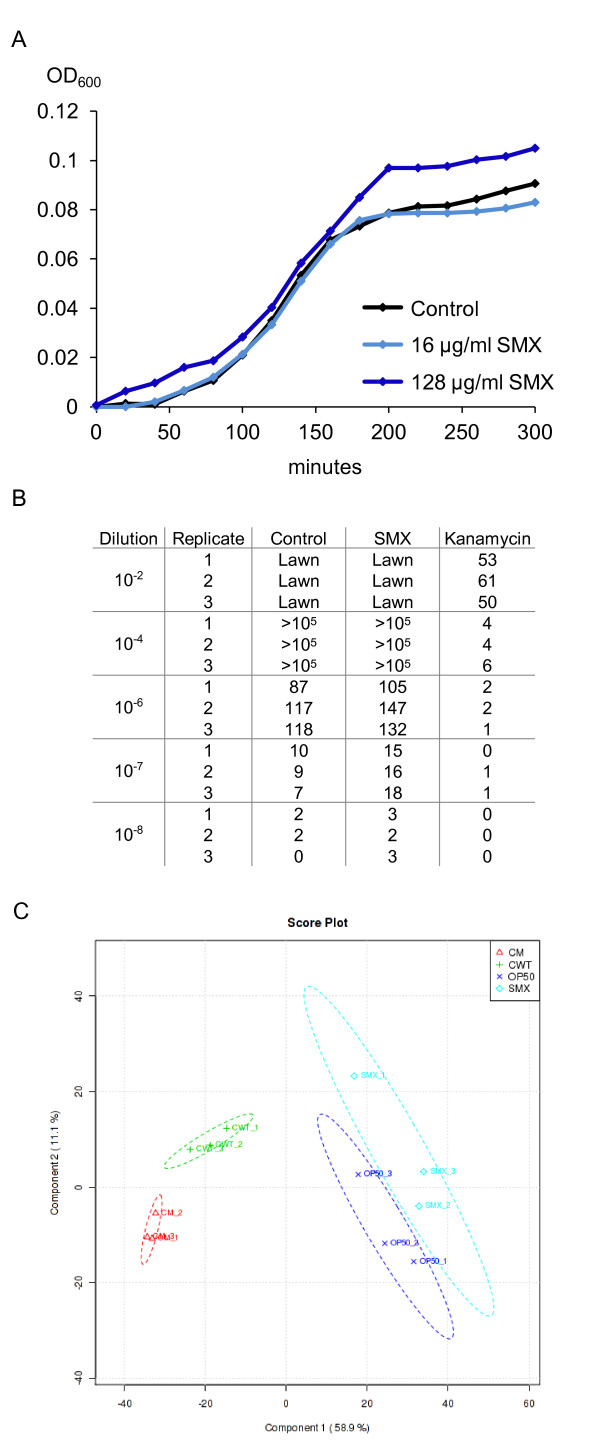
**SMX has little effect on OP50 growth, viability or metabolome**. **A**) in OD_600 _measurements of bacterial density show that SMX at 16 and 128 μg/ml has no effect on the log-phase growth rate of OP50 in liquid NGM at 37°C. **B**) Table showing colony forming units from bacteria scraped from lawns on agar plates, concentration of SMX used = 128 μg/ml. **C**) PLS-DA score plot showing two components that explain 58.9% (x-axis) and a further 11.9% (y-axis) of the variance between the conditions: whole LC-MS data from strains HT115(DE3) (labelled CWT), HT115(DE3)*aroD *(CM), OP50 (OP50) and OP50 treated with 128 μg/ml of SMX (SMX). Three replicates were performed for each condition.

SMX is known to have antibiotic properties. However, we found that in nematode growth media (NGM), concentrations of SMX that extended *C. elegans *lifespan had no significant effect on *E. coli *growth in liquid culture (Figure 4A) or on the final size of the *E. coli *lawn grown on the solid media used to culture worms (Additional file 7). These results suggest that SMX does not extend lifespan by inhibiting *E. coli *proliferation as suggested for other antibiotics [11]. To test whether SMX-treated *E. coli *encountered by worms had a decreased ability to proliferate, we tested *E. coli *lawns for cell viability. In contrast to kanamycin treatment, 128 μg/ml SMX had no effect on the colony-forming ability of OP50 (Figure 4B). Given that the folate cycle is required for cell growth, these results suggest that *E. coli *can use metabolites from the media to overcome decreased folate synthesis. In agreement with this model, *pabA *and *pabB *mutants are reported to be viable and grow normally on rich media [16,26]. Thus, there are conditions that folate synthesis can be inhibited without affecting bacterial growth and it may be that *E. coli *are adapted for such conditions in the mammalian intestine.

### Both the *aroD *mutation and SMX treatment have minor effects on metabolism

To examine the effects of SMX on broader metabolism, we re-analyzed the metabolite data from the LC-MS analysis of *E. coli *folates. We performed a global comparison between the *aroD *mutant, the HT115(DE3) wild type, OP50 and OP50 treated with 128 μg/ml SMX. A total of 1,539 features were detected, including common metabolites such as ATP, NADPH and acetyl CoA. Partial least squares discriminant analysis (PLS-DA) of the data (see Methods) shows that both the SMX and the *aroD *mutation have metabolite profiles very similar to their respective controls, having a smaller effect than the difference between the OP50 and HT115 control strains (Figure 4C). This analysis supports the hypothesis that the inhibition of folate synthesis under conditions that result in increased lifespan has only a minor effect on whole cell metabolism.

### SMX leads to a decrease in *C. elegans *folate levels without adverse effects

**Figure 5:**
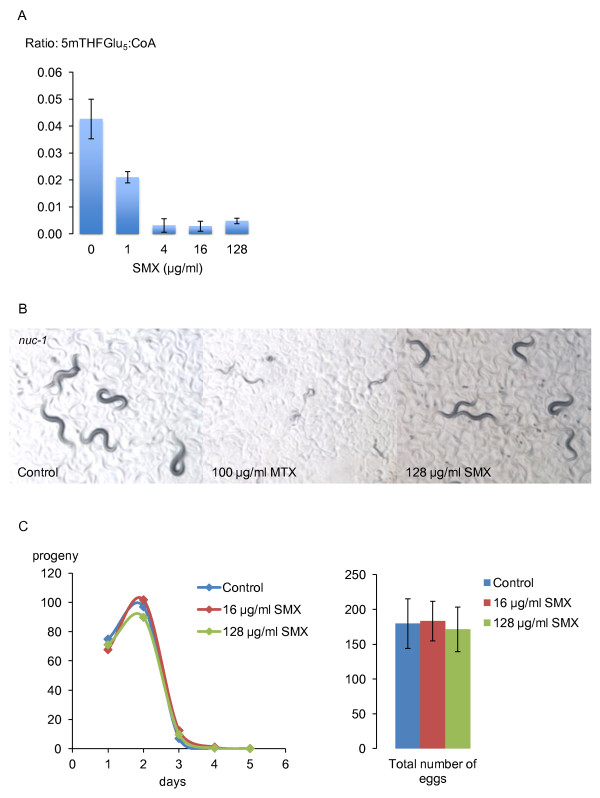
**Effects of SMX on *C. elegans *folates and folate-dependent functions**. **A**) SMX causes a dose-dependent decrease in *C. elegans *5-methylTHFGlu_5 _levels. Data shown are from three biological replicates. **B**) Methotrexate induces slowed development, sterility and uncoordination in *nuc-1 *mutant worms but 128 μg/ml SMX has no effect. Pictures were taken three days after egg-laying. **C**) 16 and 128 μg/ml SMX have no effect on development time, egg-laying schedule or total brood size of N2 worms raised at 25°C. Control (number of animals = 15), 16 μg/ml SMX (n = 18), 128 μg/ml SMX (n = 17). Error bars are ± standard deviation.

All animals must obtain folate from their food or intestinal microbes so inhibition of bacterial folate synthesis would be expected to decrease *C. elegans *folate levels. 5-methylTHFGlu_5 _was the most abundant folate species we could detect in worms. SMX decreased levels of this folate substantially but detectable levels remained (Figure 5A, Additional file 4, Methods). To test whether this decrease in folate levels led to a functional deficiency, we examined *nuc-1 *mutants, which are sensitized to methotrexate, an inhibitor of dihydrofolate reductase. Concentrations of methotrexate that have no effect on the wild-type animals cause *nuc-1 *mutants to produce sterile and uncoordinated progeny [27] (Figure 5B). However, SMX had no effect on *nuc-1 *mutants (Figure 5B), demonstrating that animals maintain a functional folate cycle. To test folate sufficiency further, we examined developmental rate and fecundity in the presence of SMX. We found that SMX had no effect on the time taken for animals to reach reproductive age or their subsequent brood size (Figure 5C). This result suggests that SMX does not impact the biosynthetic capability of the folate cycle. Further, this result confirms the drug does not interfere with *C. elegans *nutrition, or cause dietary restriction in the usual sense. However, it remains possible that life extension in *C. elegans *by dietary restriction via limitation of *E. coli*, and inhibition of microbial folate synthesis involve some common mechanisms.

Our data show that, via inhibition of bacterial folate synthesis, it is possible to slow animal aging with a minimal effect on bacterial growth. Such an intervention may allow manipulation in the mammalian gut without large disruption of the microbiota. Intriguingly, an early study showed that administration of a sulfonamide (sulfadiazine) extends the lifespan of rodents [28,29]. Moreover, as in the present study, this effect was reversed by the addition of PABA, implicating microbial folate synthesis as the target. Several species of bacteria, including *E. coli*, excrete folates, suggesting that they produce more than they need [30]. In our system, *C. elegans *is solely dependent on bacterial folate, and yet we can achieve a positive effect on lifespan without compromising healthy development, suggesting that the *C. elegans *requirement for folate is much lower than is available to them from *E. coli*. Interestingly, when added to high-folate rat food, the sulfonamide succinyl sulfathiazole, inhibited bacterial folate synthesis but had only a minor effect on rat liver folate levels [31], raising the possiblity that we can reduce folate specifically in the gut bacteria, without folate restricting the animal.

## Conclusions

Genetic or pharmacological inhibition of *E. coli *folate synthesis leads to an increase in *C. elegans *lifespan without causing detrimental effects on either the microbe or the animal. Whether a decrease in folate acts on a process in the microbe or directly in the animal or both to extend lifespan remains to be determined. However, the identification of bacterial folate synthesis as a target suggests that eliminating excessive microbial folate in the gut microbe environment could be a route to slow aging.

## Methods

### Strains

*C. elegans *strains used in this study are GA303 *rrf-3(pk 1426); daf-2(m577) *[32], GR1307 *daf-16(mgDf50) *[33], N2 (wild type), NL2099 *rrf-3(pk1426) *[34] and SS104 *glp-4(bn2) *[35].

All *E. coli *strains used in this study are listed in Table 1.

**Table 1:** *E. coli *strains

Strains	Genotype/Relevant characteristics	Source
**OP50**	OP50 *ura*	[37]
**RNAi control strain**	HT115(DE3) W3110 *rnc14::Tn10 *(DE3 PlacUV5-T7 polymerase)	[46]
***aroD *mutant**	HT115(DE3) *aroD717::IS1*	This study
**Keio collection WT**	BW25113	[16]
**Keio collection *aroD***	BW25113 *aroD*	[16,36], This study
**OP50 Su resistant**	OP50 R26	This study; [47]
***aroD *mutant/*ugt-27 *RNAi**	HT115(DE3)*aroD717::IS1/*L4440*(ugt-27)*	This study; [38]
**RNAi control + vector**	HT115(DE3)/pMMB67EH	This study
***aroD *mutant + vector**	HT115(DE3)*aroD717::IS1*/pMMB67EH	This study
**mutant + *aroD ***	HT115(DE3)*aroD717::IS1*/pMMB67EH[*aroD*]	This study
***ubiG *mutant**	GD1 *ubiG*	[15]
***ubiG *mutant + rescue**	GD1 *ubiG*/pAHG(*ubiG+*)	[15]

OP50 R26 was made in this study by mating with C600 R26 and selecting with SMX on minimal media containing uracil. As the Keio *aroD *mutant was reported to be a mixture of mutant and other strains [36], a colony was isolated and confirmed to be mutant by PCR.

### Culture conditions

NGM was prepared as described [37] using 2.5 g/l soy peptone (Product number P6713, Sigma-Aldrich Corp., Saint Louis, MO, USA) and 20 g/l high-purity agar (Sigma, Product number 05038). High purity agar is used because standard agar can give batch-to-batch variation in the *aroD *effect on lifespan, probably due to contaminating aromatic compounds. Plates were supplemented with the indicated compounds. For the kanamycin treatment of bacteria, 80 μl of 10 mM kanamycin was added after 24 hours of bacterial growth as described [11]. All compounds were from Sigma-Aldrich.

### Lifespan analysis

Unless indicated differently in Additional file 1, survival analyses were performed by the following method: eggs were prepared by bleaching adults to remove all microbes, and then placed onto plates containing either *aroD *mutant bacteria or, where relevant, SMX-treated OP50 plates. Animals were raised at 15°C until adulthood due to the temperature sensitivity of mutant phenotypes. Gravid adults were used to lay eggs onto fresh *aroD *mutant or SMX-treated OP50 plates. At L3/L4 these animals were transferred to 25°C and larvae of equivalent stage were put onto at least 5 plates of 25 worms for each condition. Animals were transferred to fresh plates after 7 and 14 days and scored for survival every 2 or 3 days. Lifespan data were analysed by JMP statistical software (SAS Institute Inc., Cary, NC, USA). Where relevant, statistical significance was determined using the Log-Rank and Wilcoxon tests of fitting to the Kaplan-Meier survival model.

### Characterisation of the life-extending effect of the mutant *E. coli *strain

All lifespan experiments conducted in this study are summarized in Additional file 1. The *E. coli *mutant was discovered because it extended the lifespan of *rrf-3(pk1426); daf-2(m577) *mutants. We then tested wild type *C. elegans *(N2), *rrf-3(pk1426) *mutants, *daf-16(mgDf50) *mutants and temperature-sensitive sterile mutants *glp-4(bn2)*, shifted from 15°C to 25°C at L4. The mutant bacteria extended lifespan of all *C. elegans *strains. The effect at 25°C was stronger than at 20°C. To test whether the mutant bacteria exerted its effect during adulthood, we shifted animals on mutant bacteria onto wild type bacteria, and *vice versa*, just before the beginning of adulthood as L4 larvae. Shifting from mutant bacteria to wild type bacteria caused *C. elegans *to have a wild type lifespan. Shifting in the other direction from wild type to mutant increased lifespan but took several days to have an effect, probably because residual wild type bacteria chemically complement the mutant bacteria with secreted PABA. These experiments imply that the effect of the bacteria on lifespan is exerted during adulthood. In all subsequent experiments, animals were raised on the mutant bacteria and then transferred to the experimental conditions at the L4 or young adult stage. Although the mutant occurred in a strain from the Ahringer RNAi library [38], the mutation occurred during culture in our laboratory. We have tested the original *ugt-27 *strain in the Ahringer library and it does not have the mutation.

### Complementation screen to identify *aroD*

To identify the mutated gene we took advantage of the inability of the mutant bacterial strain to grow on minimal media plates. We partially digested the genomic DNA of the control strain using the four-base cutter *Bfu*CI that leaves *Bam*HI compatible ends and ligated the fragments in a *Bam*HI-digested pMMB67EH, a low copy IncQ plasmid [39]. We transformed the ligation mixture into the mutant strain and screened for large colonies on minimal media plates. Untransformed dead bacteria provided enough nutrients to support the growth of small colonies of transformed mutant bacteria, allowing estimation of the numbers screened. Colonies that were clearly larger than their neighbors were picked, grown up and the plasmid isolated by mini-prep. These plasmids were retested by transformation into the mutant bacteria to test for enhanced growth on minimal media. From over 6,000 colonies, 6 independent plasmids passed this second round of screening. Sequencing of these plasmids revealed that one contained the gene *folC*, one contained *folD*, three contained *aroK *and one contained *aroD*/*ydiB*. This latter plasmid had the largest effect on mutant growth. The other plasmids appeared to have a positive effect on growth of both mutant and control strains. PCR and sequencing of the *aroD *region identified an IS1 transposon inserted in the *aroD *gene in the mutant strain but not in the control (Figure 1C). Composition of minimal media (based on ref [40]) is as follows: 15 g/l agar, 2 g/l D-glucose, 2.17 g/l Na_2_HPO_4_, 1.35 g/l KH_2_PO_4_, 0.5 g/l (NH_4_)_2_SO_4_, 0.01 g/l Ca(NO_3_)_2_, 0.005 g/l thiamine, 0.088 g/l adenine, 0.044 g/l arginine, 0.11 g/l asparagine, 0.088 g/l cysteine, 0.11 g/l glutamatic acid, 0.088 g/l histidine, 0.066 g/l isoleucine, 0.133 g/l leucine, 0.066 g/l lysine, 0.088 g/l methionine, 0.066 g/l phenylalanine, 0.422 g/l serine, 0.221 g/l threonine, 0.088 g/l tryptophan, 0.044 g/l tyrosine, 0.088 g/l uracil, 0.166 g/l valine. The composition of 1% trace element solution is 5 g/l EDTA, 0.5 g/l FeCl_3_, 0.05 g/l ZnO, 0.01 g/l CuCl_2_, 0.01 g/l CoCl_2_.6H_2_O, 0.01 g/l H_3_BO_3_.

### Folate analysis

#### *E. coli *extraction

Bacterial lawns that had been incubated at 25°C were scraped from NGM agar plates with M9 solution. The final volume of the solution multiplied by the OD_600 _of the solution diluted 1:5 gave a measure of the amount of material. The samples were concentrated by centrifugation, washed into microcentrifuge tubes and centrifuged again. The pellets were snap frozen in liquid nitrogen and resuspended in a volume of cold 80% methanol: 20% folate extraction buffer (FEB - 50 mM HEPES, 50 mM CHES, 0.5% w/v ascorbic acid, 0.2 M DTT, pH 7.85 with NaOH) in proportion to bacterial content (0.0375 × OD_600 _× original solution volume). Samples were spiked with 2:1,000 1 mg/ml methotrexateGlu_6 _as an internal standard and were sonicated on ice using a probe sonicator, centrifuged for five minutes in a cooled microcentrifuge at full speed and the supernatants were kept for analysis.

#### *C. elegans *extraction

Synchronized worms at the first day of adulthood incubated at 25°C were washed from 9 cm plates with M9 and allowed to settle. The supernatant was removed and the worms were washed with M9 and allowed to settle again to remove any remaining bacteria. Worms were then transferred to microcentrifuge tubes, gently centrifuged, the volume of pellets estimated. Worms were then washed twice into FEB and left in a total of twice the pellet volume. Proteinase K was added to a final concentration of 0.5 mg/ml and animals were then shaken vigorous at 37°C for 90 minutes. An equal volume of ice cold methanol spiked with 1:1,000 1 mg/ml methotrexateGlu_6 _was added, vortexed and centrifuged at 4°C as above.

### HPLC-MS analysis

We used methodology based on previous literature [21,22]. For HPLC, a C18 reversed phase column (Waters Acquity BEH, 100 mm × 2.1 mm (Waters Corporation, Milford, MA, USA)) was used with dimethylhexylamine as an ion-pairing reagent as in reference [21]. The mobile phase consisted of (A) methanol/water (5:95, v/v) with 5 mM dimethylhexylamine, pH 8, and (B) methanol with 5 mM dimethylhexylamine, at a flow rate of 0.2 mL/min. A linear gradient from 22% B to 80% B over nine minutes was followed by a one minute isocratic hold at 80% B. The column was then re-equilibrated for one minute at 22% B. The injection volume was 10 μl. The mass analysis was by negative mode electrospray ionisation time-of-flight (negative ESI TOF) utilizing a Q-TOF Premier instrument (Waters Corporation) calibrated with sodium formate and with dynamic correction from a leucine encephalin lockspray. Sampling cone voltage was -35 V and capillary voltage was -2,500 V. The following standards (from Schircks (Schircks Laboratories, Jona, Switzerland)) were used: 5-formylTHF (folinic acid), folic acid, 5-formylTHFGlu_3_, methotrexate, methotrexateGlu_6_. The elution times and masses were consistent between runs and corresponded to the published literature [21,22]. We fragmented the signal corresponding to 5-methylTHFGlu_5 _in the *C. elegans *sample using MS-MS and it showed the expected products. Conditions that resulted in decreased levels of signal for 5-methylTHFGlu_5 _were accompanied by similar changes in the levels of signal for the mass corresponding to 5-methylTHFGlu_4_. The peaks obtained by selecting the specific mass were integrated using MassLynx software (Waters Corporation) and used as an indication of quantity. The peaks generated by known metabolites were also integrated and the peak corresponding to coenzyme A was chosen for use in normalization because it was large and there was little variation between samples.

### Metabolomic PLS-DA

Raw LC-MS data were preprocessed with XCMS [41], a Bioconductor package for R [42]. Sample-wise normalization was done experimentally, by ensuring that all samples had a similar concentration of biological material, and feature intensity was normalized with a log transform. Both normalization and further statistical analysis, including the PLS-DA plot, were performed with MetaboAnalyst [43].

### Bacterial growth rate measurements

Liquid NGM media was aliquoted into sterile conical flasks. SMX was added to the appropriate samples and sterile water added to the control samples. OP50 was then seeded from overnight culture into each flask, and initial OD_600 _measured for each sample using liquid media as a blank. The samples were then placed on a shaker set to 200 rpm at 37°C, and OD_600 _measured every 20 minutes for 300 minutes or until growth had reached a plateau.

To measure bacterial growth on solid media, bacteria were seeded, left at room temperature for 48 hours and then transferred to 25°C for a further 24 hours. A total of 1.5 ml of M9 buffer was then added to each plate and, using a glass spreader, the bacterial lawn was scraped from the plate. The M9-containing bacteria were then pipetted into a microfuge tube. These samples were diluted 1:5 in M9 buffer for final OD_600 _measurements. This measurement was multiplied by the final volume of M9 to provide a relative measure of lawn density.

### Development and fecundity measurements

N2 worms were cultured individually from eggs at 25°C on the appropriate media. From the beginning of adulthood, animals were transferred to fresh plates every 24 hours until egg-laying stopped. The progeny from each plate was allowed to develop for two days and then counted.

## Abbreviations

CHES: N-Cyclohexyl-2-aminoethanesulfonic acid; DHF: dihydrofolate; ESI TOF: electrospray ionisation time-of-flight; FEB: folate extraction buffer; Glu: glutamate; HEPES: 2-[4-(2-hydroxyethyl)piperazin-1-yl]ethanesulfonic acid; HPLC: high pressure liquid chromatography; HPLC-MS: high pressure liquid chromatography coupled to mass spectrometry; LC-MS: liquid chromatography coupled to mass spectrometry; MS-MS: tandem mass spectrometry; NGM: nematode growth medium; OD_600_: optical density at absorbance 600 nm; PABA: para-aminobenzoic acid; PEP: phosphoenolpyruvate; PHB: para-hydroxybenzoic acid; PLS-DA: partial least squares discriminant analysis; Q-TOF: quadrupole time-of-flight; RNAi: RNA interference; SHK: shikimic acid; SMX: sulfamethoxazole; THF: tetrahydrofolate.

## Competing interests

The authors declare that they have no competing interests.

## Authors' contributions

The study was conceived by DW with contributions from DG. The experiments were designed by DW, BV and JW. The experiments were performed and analyzed by BV, DW, IF, DPD, JJ, ZB and EH. Further analysis was performed by GC. Method development was carried out by GC, NO, DPD, BV, DW and RE. The manuscript was written by DW with contributions from DG, BV and GC. Correspondence and requests for material should be addressed to DW: david.weinkove@durham.ac.uk.

All authors have read and approved the final manuscript.

## Supplementary Material

Additional file 1 **Summary of all lifespan experiments conducted in this study**. File showing individual experiments, number of animals scored as dead, number censored and, where relevant, percent increase in lifespan between mutant and control, and *P*-values from Log-Rank and Wilcoxon tests of the Kaplan-Meier survival model. Click here for file

Additional file 2 **Spontaneous *E. coli *mutant rather than RNAi plasmid extends *C. elegans *lifespan**. **A**) A new RNAi strain containing the *ugt-27 *plasmid has no effect on lifespan. Survival of *rrf-3 *worms (20°C) on the control HT115(DE3) strain with the L4440 plasmid, (control, n = 61), the original *ugt-27 *strain (mutant, n = 98) and a new strain consisting of HT115(DE3) transformed with the *ugt-27 *plasmid (new, n = 50). Difference between mutant and control, 29.3% (*P *= < 0.0001). Click here for file

Additional file 3 **PABA supplementation has no toxic effect**. **A**) PABA supplementation reverses the lifespan extension on the mutant bacteria (*aroD *+ PABA, n = 85, *aroD*, n = 124), but has no effect on the control bacteria (control + PABA, n = 112, control, n = 84). **B**) Addition of PABA has no effect on *ubiG *bacteria (*ubiG *+ PABA, n = 90, *ubiG*, n = 76) or *ubiG- *+ rescue plasmid (*ubiG:pAHG *+ PABA, n = 116, *ubiG:pAHG*, n = 118). **C**) PHB supplementation had no effect of lifespan of *C. elegans *maintained on either the mutant (*aroD*, n = 161, *aroD *+ 25 μM PHB, n = 129, *aroD *+ 250 μM, n = 105) or control bacteria (control, n = 173, control + 25 μM PHB, n = 121, control + 250 μM PHB, n = 130). See Additional file 1 for a full listing of all lifespan data in this study. Click here for file

Additional file 4 **Relevant traces from the HPLC/MS analysis from *E. coli***. **A**) Traces from HT115(DE3) and HT115(DE3)*aroD *of *m/z *= -730.244 corresponding to the negative ion of formylTHFGlu_3_. **B**) Traces from formylTHFGlu_3 _show that this species becomes undetectable in OP50 with increasing concentrations of SMX. **C**) Traces from *C. elegans *lysates corresponding to the negative ion of 5-methylTHFGlu_5_, with 0, 0.1 and 1 μg/ml SMX. Click here for file

Additional file 5 **Effect of media supplementation with folate on *C. elegans *lifespan on *aroD *and *ubiG *mutants**. (**A**) *glp-4(bn2) *animals were raised on the *aroD *mutant until L4 (15°C) and then transferred (25°C) to the *aroD *mutant (n = 100), control (n = 126), *aroD *+ 1 × folate (n = 101), *aroD *+ 2 × folate (n = 149). *aroD *vs *aroD *+ 1 × folate, *P *= < 0.0001; *aroD *+ 1 × folate vs *aroD *+ 2 × folate, *P *= 0.13 (Log Rank), *P *= 0.055 (Wilcoxon). (**B**) *glp-4 *worms were raised on *E. coli *OP50 (15°C) and transferred (25°C) at L4 to *ubiG *(n = 145), *ubiG *+ pAHG (*ubiG^+ ^*rescue plasmid), GD1 + 1 × folate (n = 145), GD1+ 2 × folate (n = 146). (**C**) Traces from the HPLC/MS analysis of the wild type (HT115(DE3)) and *aroD *mutant (HT115(DE3)*aroD*) bacteria. Peaks shown are *m/z *= 730.25 and 548.67 corresponding to formylTHFGlu_3 _and the methotrexateGlu_6 _spiked standard respectively with increasing folic acid supplementation (1 × folate is equal to 294 μM folic acid). Click here for file

Additional file 6 **PABA reverses the lifespan increase caused by 16 μg/ml and 128 μg/ml SMX**. Lifespan curves showing Control (n = 102), 16 μg/ml SMX (n = 176), 128 μg/ml SMX (n = 229), 16 μg/ml SMX + 250 μM PABA (n = 160), 128 μg/ml SMX + 250 μM PABA (n = 217). Click here for file

Additional file 7 **Lawn density of plates treated with various concentrations of SMX**. Relative bacterial content of lawns from the mean values from 10 plates per conditions (see Methods). Error bars are ± standard deviations. Student's *t*-test values (Control vs 8 μg/ml SMX: *P *= 0.02, Control vs 16 μg/ml: *P *= 0.04, Control vs 64 μg/ml: *P *= 0.12, Control vs 128 μg/ml: *P *= 0.04). Click here for file
