# Supplementary figures and images for: Excessive folate synthesis limits lifespan in the C. elegans: E. coli aging model
Source: BMC Biol. 2012 Jul 31;10:67. doi: 10.1186/1741-7007-10-67 (PMC3583181; doi:10.1186/1741-7007-10-67)

Virk *et al.* Additional File 2

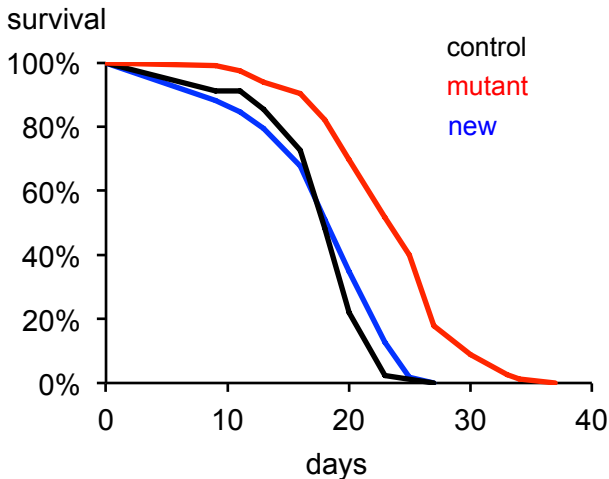

Supplement: Additional file 2 — Spontaneous E. coli mutant rather than RNAi plasmid extends C. elegans lifespan. A) A new RNAi strain containing the ugt-27 plasmid has no effect on lifespan. Survival of rrf-3 worms (20°C) on the control HT115(DE3) strain with the L4440 plasmid, (control, n = 61), the original ugt-27 strain (mutant, n = 98) and a new strain consisting of HT115(DE3) transformed with the ugt-27 plasmid (new, n = 50). Difference between mutant and control, 29.3% (P = < 0.0001). [file 1741-7007-10-67-S2.pdf]

A

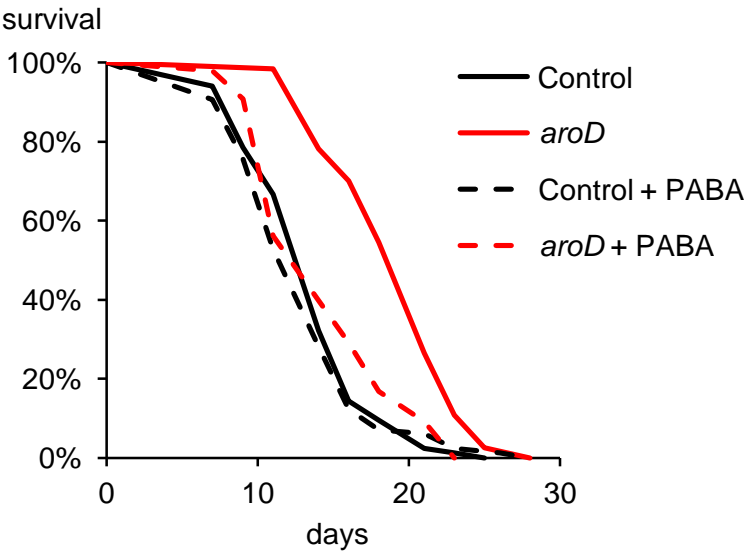

B

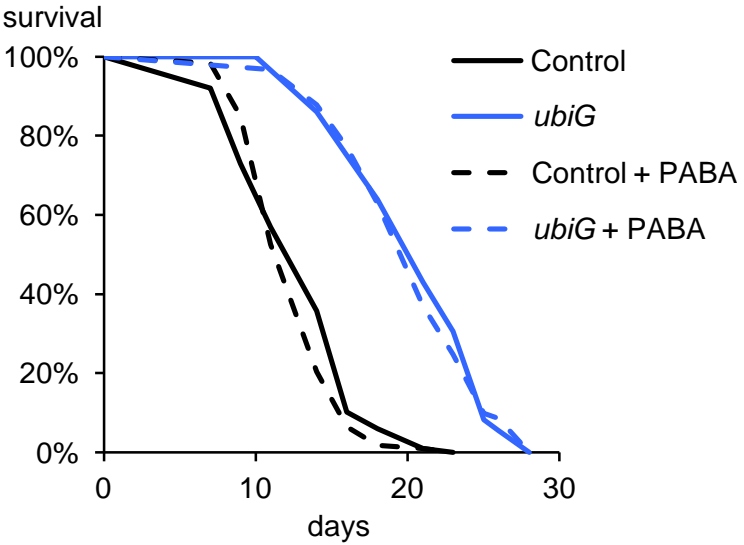

C

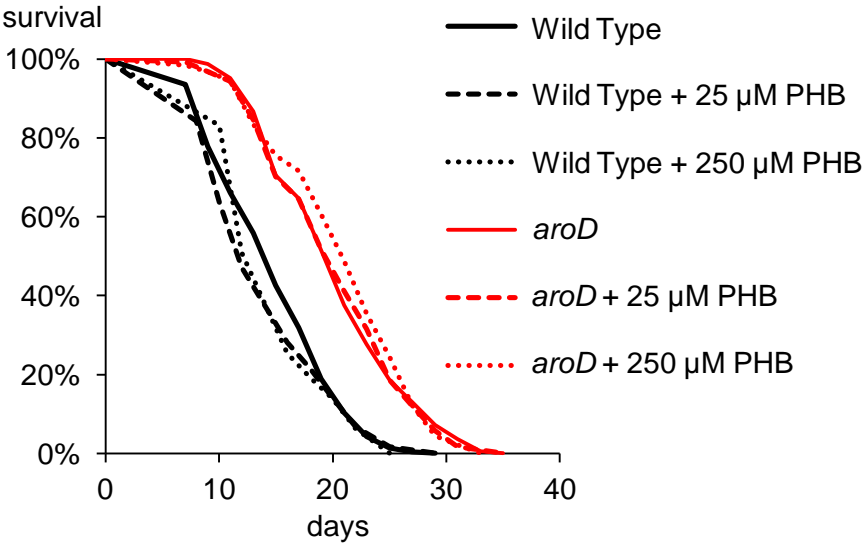

Supplement: Additional file 3 — PABA supplementation has no toxic effect. A) PABA supplementation reverses the lifespan extension on the mutant bacteria (aroD + PABA, n = 85, aroD, n = 124), but has no effect on the control bacteria (control + PABA, n = 112, control, n = 84). B) Addition of PABA has no effect on ubiG bacteria (ubiG + PABA, n = 90, ubiG, n = 76) or ubiG- + rescue plasmid (ubiG:pAHG + PABA, n = 116, ubiG:pAHG, n = 118). C) PHB supplementation had no effect of lifespan of C. elegans maintained on either the mutant (aroD, n = 161, aroD + 25 μM PHB, n = 129, aroD + 250 μM, n = 105) or control bacteria (control, n = 173, control + 25 μM PHB, n = 121, control + 250 μM PHB, n = 130). See Additional file 1 for a full listing of all lifespan data in this study. [file 1741-7007-10-67-S3.pdf]

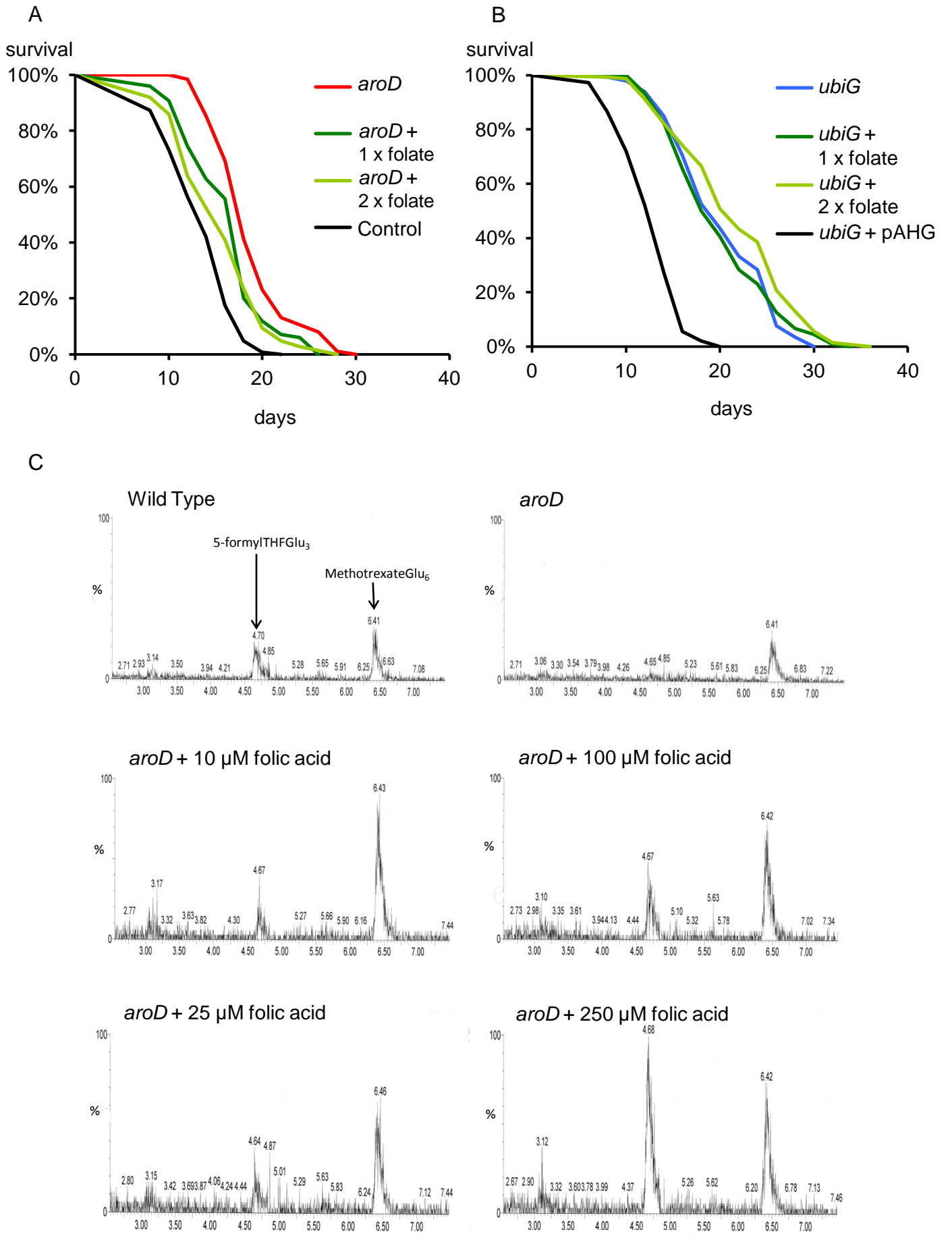

Supplement: Additional file 5 — Effect of media supplementation with folate on C. elegans lifespan on aroD and ubiG mutants. (A) glp-4(bn2) animals were raised on the aroD mutant until L4 (15°C) and then transferred (25°C) to the aroD mutant (n = 100), control (n = 126), aroD + 1 × folate (n = 101), aroD + 2 × folate (n = 149). aroD vs aroD + 1 × folate, P = < 0.0001; aroD + 1 × folate vs aroD + 2 × folate, P = 0.13 (Log Rank), P = 0.055 (Wilcoxon). (B) glp-4 worms were raised on E. coli OP50 (15°C) and transferred (25°C) at L4 to ubiG (n = 145), ubiG + pAHG (ubiG+ rescue plasmid), GD1 + 1 × folate (n = 145), GD1+ 2 × folate (n = 146). (C) Traces from the HPLC/MS analysis of the wild type (HT115(DE3)) and aroD mutant (HT115(DE3)aroD) bacteria. Peaks shown are m/z = 730.25 and 548.67 corresponding to formylTHFGlu3 and the methotrexateGlu6 spiked standard respectively with increasing folic acid supplementation (1 × folate is equal to 294 μM folic acid). [file 1741-7007-10-67-S5.pdf]

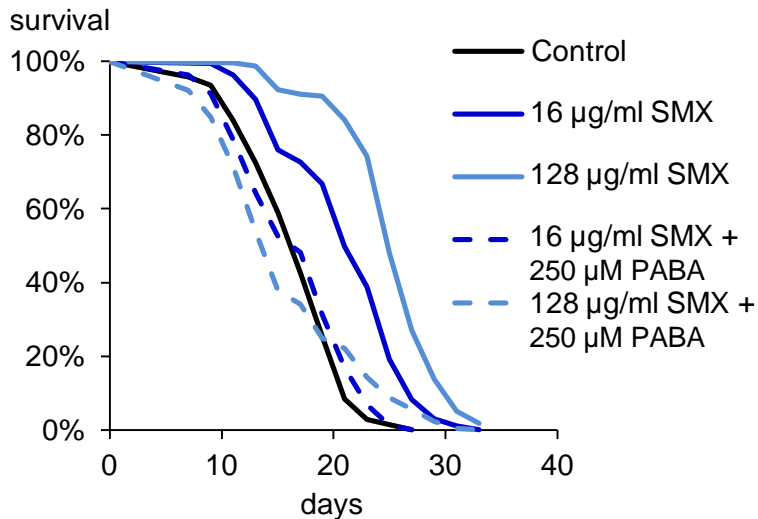

Supplement: Additional file 6 — PABA reverses the lifespan increase caused by 16 μg/ml and 128 μg/ml SMX. Lifespan curves showing Control (n = 102), 16 μg/ml SMX (n = 176), 128 μg/ml SMX (n = 229), 16 μg/ml SMX + 250 μM PABA (n = 160), 128 μg/ml SMX + 250 μM PABA (n = 217). [file 1741-7007-10-67-S6.pdf]

Lawn density (% OP50)

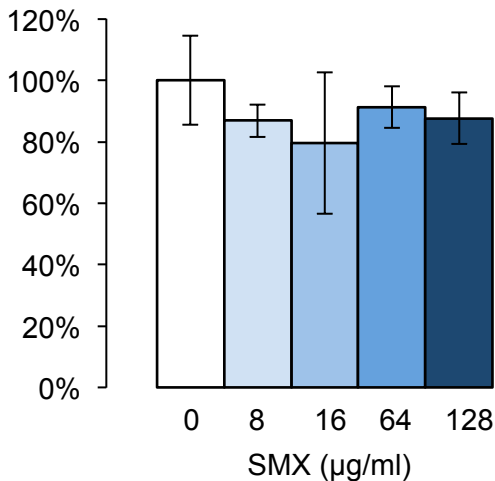

Supplement: Additional file 7 — Lawn density of plates treated with various concentrations of SMX. Relative bacterial content of lawns from the mean values from 10 plates per conditions (see Methods). Error bars are ± standard deviations. Student's t-test values (Control vs 8 μg/ml SMX: P = 0.02, Control vs 16 μg/ml: P = 0.04, Control vs 64 μg/ml: P = 0.12, Control vs 128 μg/ml: P = 0.04). [file 1741-7007-10-67-S7.pdf]
