# Supplementary material for: Excessive folate synthesis limits lifespan in the C. elegans: E. coli aging model
Source: BMC Biol. 2012 Jul 31;10:67. doi: 10.1186/1741-7007-10-67 (PMC3583181; doi:10.1186/1741-7007-10-67)
Supplement: Additional file 4 — Relevant traces from the HPLC/MS analysis from E. coli. A) Traces from HT115(DE3) and HT115(DE3)aroD of m/z = -730.244 corresponding to the negative ion of formylTHFGlu3. B) Traces from formylTHFGlu3 show that this species becomes undetectable in OP50 with increasing concentrations of SMX. C) Traces from C. elegans lysates corresponding to the negative ion of 5-methylTHFGlu5, with 0, 0.1 and 1 μg/ml SMX. [file 1741-7007-10-67-S4.pdf]

Virk *et al.* Additional File 4

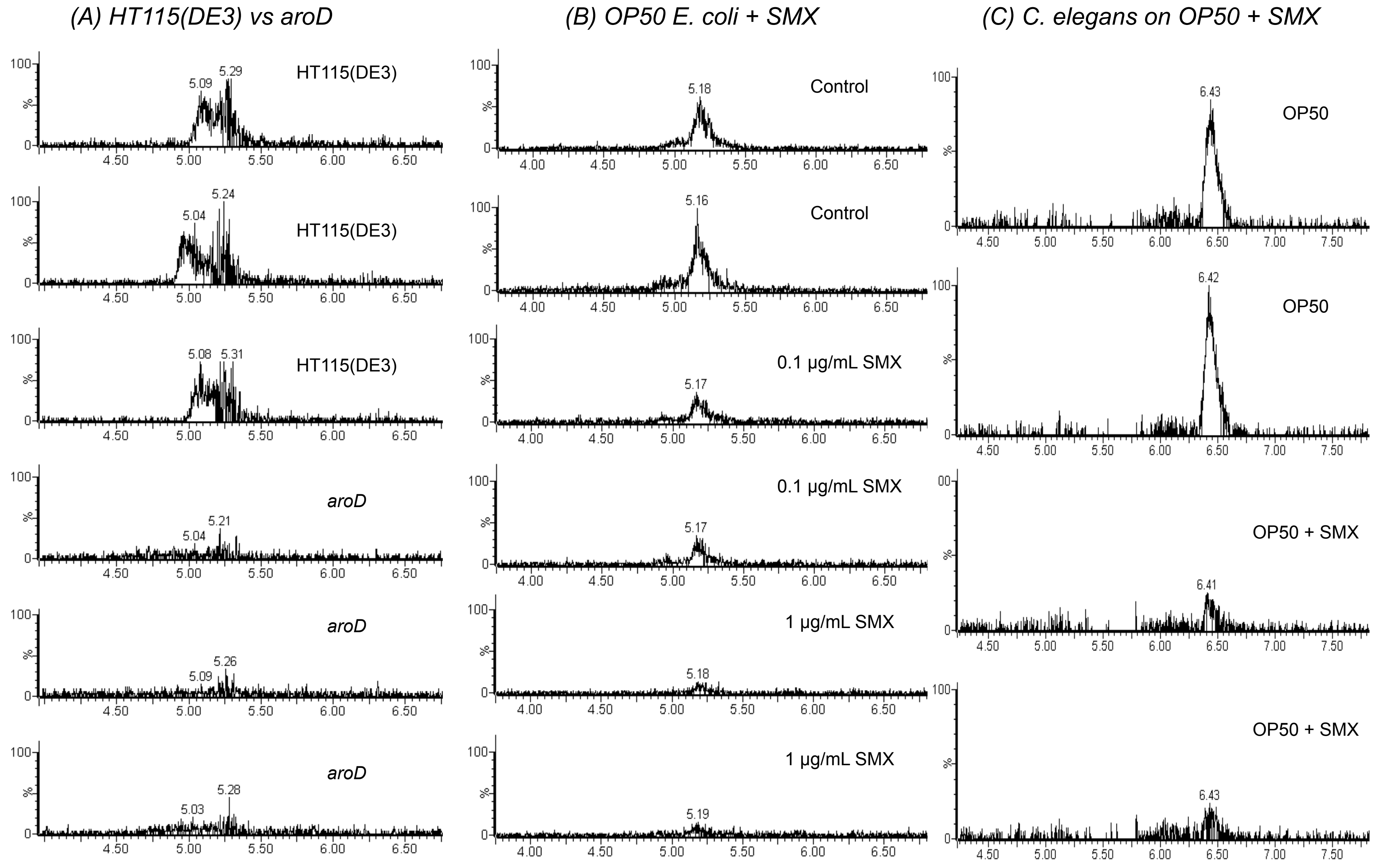

(A) Comparison of formylTHFGlu$_3$ levels on HT115(DE3) and HT115(DE3)*aroD E. coli*, 3 biological replicates each (*m/z* = -730.244 ± 0.1 Da)

(B) Traces of formylTHFGlu$_3$ levels in OP50 *E. coli* and OP50 treated with 0.1 µg/ml and 1 µg/ml SMX, 2 biological replicates each (*m/z* = -730.244 ± 0.1 Da)

(C) HPLC-MS traces of 5-methylTHFGlu$_5$ levels in *C. elegans* grown on OP50 *E. coli* and OP50 plus 128 µg/ml SMX, 2 biological replicates for each condition (*m/z* = -974.344 ± 0.1 Da)
